# Nogo-related frontal theta-band activity after magnetic seizure therapy in treatment-resistant depression: an exploratory task-based EEG study

**DOI:** 10.3389/fpsyt.2026.1950544

**Published:** 2026-10-28

**Authors:** Lu Yuan, Fan Chang, Chenghui Yang, Cui Yuan, Bo Zhou, Yuejia Luo, Ruifeng Shi, Yihan Su, Xue Xia, Ying He

**Affiliations:** 1 Sichuan Provincial Center for Mental Health, The Center of Psychosomatic Medicine of Sichuan Provincial People's Hospital, University of Electronic Science and Technology of China, Chengdu, China; 2 Key Laboratory of Psychosomatic Medicine, Chinese Academy of Medical Sciences, Chengdu, China; 3 The State Key Lab of Cognitive and Learning, Faculty of Psychology, Beijing Normal University, Beijing, China; 4 Institute for Neuropsychological Rehabilitation, University of Health and Rehabilitation Sciences, Qingdao, China; 5 School of Psychology, South China Normal University, Guangzhou, China; 6 Faculty of Health and Wellness, City University of Macau, Macao, Macao SAR, China; 7 School of Social Development, University of Health and Rehabilitation Sciences, Qingdao, China

**Keywords:** EEG microstates, magnetic seizure therapy, response inhibition, theta-band activity, treatment-resistant depression

## Abstract

**Introduction:**

Magnetic seizure therapy (MST) is an emerging convulsive treatment for treatment-resistant depression (TRD) with efficacy comparable to electroconvulsive therapy and superior cognitive safety. However, its neurophysiological mechanisms—particularly those underlying conflict monitoring and response inhibition—remain unclear. Frontal midline theta-band activity indexes anterior cingulate–prefrontal cognitive-control activity and may reflect inefficient neural processing in depression.

**Methods:**

In this open-label, single-arm trial, 19 patients with TRD (16 female; mean age 20.89 ± 8.16 years; 10 of the 19 participants were minors) received 6–12 MST sessions using 100-Hz double-cone coil stimulation targeting bilateral F3/F4 frontal regions. Clinical assessments, including the 24-item Hamilton Depression Rating Scale (HAMD-24), Hamilton Anxiety Rating Scale (HAMA), Montgomery-Åsberg Depression Rating Scale (MADRS), and 64-channel Go/Nogo task-based electroencephalography (EEG), were performed before and after treatment. EEG outcome measures included event-related potentials (N2/P3), time-frequency power (theta/beta), microstate dynamics, and theta–beta cross-frequency coupling.

**Results:**

Following MST, patients showed significant reductions in depressive symptoms (HAMD: 40.53 ± 10.25 to 22.53 ± 11.21; MADRS: 35.68 ± 8.84 to 20.68 ± 11.12). Response rates ranged from 42.1% to 57.9%. Behavioral data could not be formally analyzed due to incomplete data storage. At an uncorrected threshold, total Nogo-related theta-band activity (4–7 Hz) at Fz and FCz was lower after treatment; however, these effects did not survive FDR correction. ERP amplitudes did not change significantly. Exploratory microstate analysis showed nominal changes in Map4 AUC during Go processing and Map2 duration during Nogo processing; none survived FDR correction. These parameters should not be interpreted as reflecting changes in neural activation or inhibitory preparation. No significant changes were observed in theta–beta cross-frequency coupling or Go-condition theta power.

**Discussion:**

These preliminary findings suggest that MST was associated with short-term symptom improvement and lower total Nogo-related theta-band activity at an uncorrected threshold. Because no control group, complete behavioral data, normative comparison, Time × Condition interaction, or EEG-clinical association was available, these findings cannot establish causal mechanisms, condition specificity, improved neural efficiency, or biomarker utility. Replication in adequately powered controlled studies with complete behavioral and longitudinal clinical measures is required.

## Introduction

Major depressive disorder (MDD) is a leading cause of global disease burden; approximately 30% of patients do not achieve remission after standard treatments and progress to treatment-resistant depression (TRD) (1). For severe TRD, convulsive therapies remain among the most effective biological treatments. Electroconvulsive therapy (ECT) is efficacious but limited by cognitive adverse effects, particularly postictal disorientation and autobiographical memory impairment. Magnetic seizure therapy (MST) was developed as a focal convulsive neuromodulation technique that induces seizures under anesthesia via high-intensity transcranial magnetic stimulation (2–4). Compared with ECT, MST produces more spatially restricted cortical stimulation and is associated with faster reorientation and fewer cognitive adverse effects (5–7).

Accumulating evidence supports MST’s antidepressant efficacy in TRD. Early open-label studies reported clinically meaningful response rates (8), and subsequent studies extended these findings to bipolar and other mood disorders with limited cognitive impairment (9). Randomized evidence indicates that MST may achieve antidepressant outcomes comparable to ultrabrief right unilateral ECT, with advantages in postictal recovery and memory preservation (10). A systematic review of 15 studies (300 participants) reported variable but clinically relevant response rates (11). In younger patients, MST may provide antidepressant benefits comparable to modified ECT with less cognitive impairment (12). However, the neurophysiological mechanisms by which MST influences cognitive-control processes in TRD remain unclear.

Cognitive dysfunction is a core feature of MDD (13). Deficits in executive function, conflict monitoring, and response inhibition often persist beyond mood symptoms and are associated with poor functional outcomes, relapse risk, and suicidal vulnerability (14). Response inhibition is particularly relevant for TRD because impaired inhibitory control may reflect fronto-cingulate dysfunction. The Go/Nogo task probes response inhibition and conflict monitoring, engaging a distributed network including the anterior cingulate cortex (ACC), dorsolateral prefrontal cortex (DLPFC), and inferior parietal regions (15). In depression, Go/Nogo performance and task-evoked electrophysiological responses are frequently altered, including N2/P3 abnormalities and atypical oscillatory activity (16, 17). Thus, Go/Nogo EEG indices may provide sensitive markers of fronto-cingulate inhibitory-control dysfunction in depression.

Among task-evoked EEG measures, frontal midline theta-band activity is especially relevant to cognitive control. Frontal theta (typically 4–7 Hz) has been implicated in conflict monitoring, error detection, and adaptive recruitment of top-down control. Source-level and intracranial evidence links frontal midline theta to the ACC and dorsomedial prefrontal cortex (dmPFC), which detect conflict and coordinate control through prefrontal and parietal networks (18). During Go/Nogo tasks, successful inhibition is often accompanied by transient increases in frontal theta power, reflecting mobilization of control processes. However, abnormal theta responses in depression may indicate inefficient or compensatory recruitment rather than efficient inhibition. In particular, in depressed patients with suicidal ideation, this theta response becomes hypersynchronized—interpreted as inefficient compensatory recruitment rather than effective inhibition (19, 20). Frontal theta activity has also been linked to treatment outcome in depression (21). Thus, treatment-related changes in theta dynamics may provide a neurophysiological index of how MST affects fronto-cingulate control processing.

Convulsive therapies can induce broad electrophysiological and network-level changes, including alterations in EEG spectral activity, functional network topology, corticothalamic seizure dynamics, and fronto-cingulate/limbic circuitry (22). Most previous MST studies have focused on clinical symptoms, neuropsychological outcomes, resting-state EEG, seizure characteristics, or TMS-EEG indices. Task-based EEG paradigms may offer additional mechanistic value by assessing neural responses under specific cognitive demands rather than during unconstrained rest (23). This is particularly important for TRD, where executive dysfunction may emerge only when patients must resolve conflict or inhibit prepotent responses. To date, few studies have systematically examined whether MST modulates task-evoked neural dynamics during inhibitory control in TRD.

The present study therefore investigated the effects of MST on Go/Nogo task-based EEG markers of inhibitory control in young patients with TRD. We combined event-related potentials (ERP), event-related spectral perturbations (ERSP), EEG microstate analysis, and cross-frequency coupling (CFC) to characterize MST-related changes across multiple temporal and spectral levels. We hypothesized that MST would be associated with reductions in depressive symptoms and with changes in Go/Nogo-related EEG indices, including total theta-band activity and microstate dynamics. These analyses were exploratory and not designed to test normalization, neural efficiency, or condition specificity. To integrate these measures, we adopted a multi-level framework. Conventional Go/Nogo ERP components (N2, P3) index phase-locked postsynaptic activity associated with conflict monitoring and inhibitory evaluation (15, 16). However, averaged ERPs primarily capture time-locked responses and may not fully reflect non-phase-locked or network-level dynamics. In contrast, frontal midline theta-band activity reflects event-related spectral perturbations linked to ACC/dmPFC-mediated conflict monitoring and cognitive control recruitment (18, 19). Since MST may alter corticothalamic and fronto-limbic network excitability (22, 24), we expected task-evoked theta to reveal information beyond conventional N2/P3 measures by indexing total event-related theta-band activity during response inhibition, without assuming genuine oscillatory synchronization. Importantly, because the 4–7 Hz analysis window contained fewer than two full theta cycles, theta findings are interpreted as changes in total theta-band activity rather than pure oscillatory amplitude (25). EEG microstates capture millisecond-scale whole-brain state organization and may complement spectral measures by describing the temporal structure of task-evoked brain states (26, 27). However, task-evoked microstates are data-driven and lack one-to-one functional assignments; microstate findings were therefore treated as exploratory. Theta–beta cross-frequency coupling indexes phase–amplitude coordination between frontal theta and central beta, a mechanism implicated in response inhibition (28, 29); we therefore restricted this exploratory analysis *a priori* to Fz theta–Cz beta during Nogo trials. These measures were considered complementary but not interchangeable. In particular, we did not assume that microstate parameters reflect the same neural process as theta power, and their relationship was not directly tested in the present study.

## Materials and methods

### Study design

This single-arm, within-subject, pre–post study examined clinical and task-evoked EEG changes before and after MST in patients with TRD. Clinical assessments and Go/Nogo EEG recordings were performed at baseline (within 24 hours before the first MST session) and 24–48 hours after the final session. This window was chosen to avoid acute postictal effects while capturing early treatment-related changes.

### Participants

Nineteen patients with TRD (16 female, 3 male; mean age 20.89 ± 8.16 years, range 13–40) were enrolled. All participants met DSM-5 criteria for major depressive disorder. TRD was defined as non-response to at least two adequate antidepressant trials from different pharmacological classes. Exclusion criteria included schizophrenia, bipolar disorder, substance use disorder, neurological disease, intracranial metal implants, or other contraindications to MST or anesthesia. All adult participants provided written informed consent. Participants under 18 years of age provided written assent after the study procedures had been explained in age-appropriate language; their parents or legal guardians provided written informed consent. The study was approved by the Institutional Review Board (Review No. 361 of 2025). Detailed individual-level data on demographics, MST treatment, anesthesia, and medication are provided in **Supplementary Table S1**; participant IDs were recoded to reduce re-identification risk.

### Concomitant medications

Concomitant psychotropic medications were maintained at stable doses for at least 2 weeks before baseline and throughout the MST course. No medication changes occurred during treatment. Detailed medication regimens are provided in **Supplementary Table S1**. Because of the limited sample size, sensitivity analyses for medication effects were not performed; this is acknowledged as a limitation.

### MST protocol

MST was administered using a YIRUIDE NS 7000 magnetic stimulator with a double-cone coil positioned over the vertex, targeting bilateral F3/F4 frontal regions. Stimulation was delivered at 100 Hz, 100% maximum device output, with a 10-second train duration. Treatments were given five times weekly, with each participant receiving 6–12 sessions (mean 8.2 ± 1.9) based on clinical response and tolerability. All sessions were performed under general anesthesia using standard modified electroconvulsive therapy procedures. Anesthesia was induced with propofol (1–2 mg/kg) and muscle relaxation was achieved with succinylcholine (0.5–1 mg/kg). Seizure activity was monitored by EEG during each session. An adequate seizure was defined as EEG seizure activity lasting longer than 10 seconds.Seizure duration was recorded for each MST session. For each participant, we calculated the mean, median, standard deviation, first-session duration, last-session duration, total duration, slope across sessions, and last-minus-first difference. These seizure duration characteristics are summarized in **Supplementary Table S2**.

### Clinical assessment

Clinical symptoms were assessed at baseline and 24–48 hours after the final MST session. Depressive symptoms were evaluated using the 24-item Hamilton Depression Rating Scale (HAMD-24) and the Montgomery-Åsberg Depression Rating Scale (MADRS) (30); anxiety symptoms were assessed using the Hamilton Anxiety Rating Scale (HAMA). Clinical response was defined as a ≥50% reduction from baseline on the depression severity scale. Remission was defined using established scale-specific thresholds: HAMD-24 ≤7, HAMA ≤7, and MADRS ≤10.

### Go/Nogo task

A classic visual Go/Nogo task assessed response inhibition. The task comprised two blocks. In the first block, the digits “1” (Go) and “9” (Nogo) were presented in a 3:1 ratio (240 trials). In the second block, the digits were reversed (“9” as Go, “1” as Nogo), also in a 3:1 ratio (240 trials). Each trial began with a fixation cross (500 ms), followed by stimulus presentation (200 ms) and a blank screen (1300 ms). Participants were instructed to respond as quickly as possible to Go stimuli and withhold responses to Nogo stimuli. Behavioral responses (reaction times for Go trials, accuracy for Go/Nogo trials, and commission errors for Nogo trials) were recorded simultaneously with EEG.

### EEG data acquisition and preprocessing

EEG data were recorded using a 64-channel Neuroscan system at 1000 Hz, with electrodes placed according to the extended international 10–20 system (31). Task-based EEG recordings were acquired within 24 hours before the first MST session and 24–48 hours after the final session. Preprocessing was performed using EEGLAB v2021.0 and the FieldTrip toolbox (32) and included removal of irrelevant electrodes; bandpass filtering at 0.5–45 Hz and notch filtering at 48–52 Hz; segmentation (–500 to 1000 ms); baseline correction using the –500 to 0 ms interval; spherical spline interpolation for bad channels; independent component analysis (ICA) to remove ocular and muscle artifacts (33); re-referencing to the global average; and rejection of trials with amplitudes exceeding ±100 μV. Data were then downsampled to 500 Hz for further analysis. One participant’s EEG data were corrupted due to technical issues and excluded from all EEG analyses, leaving a final sample of 18 participants.

### EEG measures

Event-Related Potentials (ERP). Data were re-segmented from –200 to 1000 ms relative to stimulus onset and baseline-corrected using the –200 to 0 ms interval. Mean amplitudes were extracted for Go-N2 (Fz/FCz, 220–330 ms), Go-P3 (Pz, 200–600 ms), Nogo-N2 (Fz/FCz, 220–330 ms), and Nogo-P3 (Cz, 400–700 ms). Difference waves (Nogo minus Go) were computed as N2d (FCz, 220–330 ms) and P3d (Cz, 400–700 ms).

Event-Related Spectral Perturbation (ERSP). Time-frequency decomposition used a Hanning window (250 ms, 2 ms step) with FFT over 4–45 Hz. Baseline correction used the –350 to –150 ms prestimulus interval, which differs from the ERP baseline (–200 to 0 ms) to avoid contamination by immediate pre-stimulus activity (25); this discrepancy limits direct ERP–ERSP comparability. This measure reflects total power (phase-locked and non-phase-locked), and the analysis windows (e.g., 0.16–0.40 s at 4–7 Hz) contain fewer than two full theta cycles, limiting distinction between oscillatory activity and broadband/evoked components. Results should therefore be interpreted as changes in total theta-band activity rather than pure oscillatory amplitude (25).

Microstate Analysis. Microstate analysis was performed using the RAGU toolbox. The optimal number of microstate prototypes was determined by a cross-validation procedure: the available ERP data were randomly divided into training and test sets, with 18 participants randomly split into two groups of 9; 250 cross-validations were performed, testing 3 to 10 microstate classes. k-means clustering with 50 random initializations was used to identify each microstate map. The final microstate maps were computed using the grand-average ERP across all participants and conditions. These group-level maps were then fitted to the grand-average ERP of each experimental condition (Go and Nogo, pre- and post-treatment) to extract condition-level microstate parameters, including onset, offset, duration, area under the curve (AUC), center of gravity (COG), and mean GFP. Group-level clustering (k-means) on the grand-average ERP was followed by bootstrap resampling (1000 iterations, mean similarity >0.90) to assess stability. Although originally developed for resting-state data, microstate analysis has been increasingly applied to task-evoked EEG (26, 27). Functional labels should be considered tentative because microstate numbering is dataset-dependent and task-evoked microstates do not have one-to-one functional assignments (26, 27). These labels are descriptive only and should not be interpreted as evidence of neural efficiency or inhibitory preparation. 

Cross-Frequency Coupling (CFC). We quantified cross-frequency coupling between theta phase (4–7 Hz) at Fz and beta amplitude (13–30 Hz) at Cz using the Kullback–Leibler modulation index (KLMI) with a multitaper convolution method (250-ms window, bin = 18, 200 randomizations) (28). This electrode pair was selected *a priori* based on evidence linking frontal theta and central beta activity to response inhibition (28, 29). The analysis was restricted to the Nogo condition to focus specifically on inhibitory processing rather than conducting a comprehensive whole-brain CFC analysis. Because this exploratory analysis was not corrected for multiple comparisons, the null CFC finding should be interpreted with caution.

### Statistical analysis

Statistical analyses covered clinical and EEG outcomes. Normality was assessed using the Shapiro–Wilk test. Clinical data were analyzed using paired-samples t-tests for normally distributed variables or Wilcoxon signed-rank tests otherwise. ERP, ERSP, and CFC measures were compared using paired-samples t-tests. Microstate parameters were assessed using nonparametric randomization tests (10, 000 permutations). Electrode selections (Fz, FCz, Cz, Pz) were based on *a priori* hypotheses from prior Go/Nogo studies (18, 19).To address multiplicity, two prespecified outcome families were defined: (i) ERSP: all predefined time-frequency windows; and (ii) microstate: all extracted parameters within each condition. Benjamini–Hochberg FDR was applied within each family. ERP and CFC were not included in the FDR correction because no comparison in these measures reached the uncorrected threshold. Because no EEG finding survived FDR correction, all EEG findings are reported as nominal, uncorrected, exploratory results and should be interpreted as hypothesis-generating (34).Effect sizes (Cohen’s d) are reported for all comparisons. A two-sided P < 0.05 was considered nominally significant.

## Results

### Clinical efficacy

Following MST, significant reductions were observed across all clinical symptom scales. HAMD-24 scores decreased from 40.53 ± 10.25 at baseline to 22.53 ± 11.21 after treatment, t(18) = 7.206, P < 0.001, Cohen’s d = 1.653. HAMA scores decreased from 28.53 ± 8.00 to 16.74 ± 7.69, t(18) = 5.575, P < 0.001, d = 1.279. MADRS scores decreased from 35.68 ± 8.84 to 20.68 ± 11.12, t(18) = 7.419, P < 0.001, d = 1.702. These large within-subject effect sizes indicate substantial acute reductions in depressive and anxiety symptoms after MST (**Table 1**).

**Table 1:** Paired t-test results for pre- and post-treatment rating scale scores.

Scale	Method	Shapiro-Wilk p	Pre-treatment (mean ± SD)	Post-treatment (mean± SD)	t(18)	p	Cohen’s d
HAMD	Paired t-test	0.7284	40.53 ± 10.25	22.53 ± 11.21	7.206	<0.001	1.653
HAMA	Paired t-test	0.9376	28.53 ± 8.00	16.74 ± 7.69	5.575	<0.001	1.279
MADRS	Paired t-test	0.2663	35.68 ± 8.84	20.68 ± 11.12	7.419	<0.001	1.702

Shapiro-Wilk tests were performed on paired differences. All p values were >0.05, indicating that the paired differences were normally distributed; paired-samples t-tests were therefore used. Cohen’s d was calculated based on paired differences. df = 18. SD, standard deviation.

With a ≥50% reduction from baseline, response rates were 42.1% for HAMD-24 (8/19), 42.1% for HAMA (8/19), and 57.9% for MADRS (11/19). Remission rates were lower: 0% for HAMD-24, 5.3% for HAMA (1/19), and 10.5% for MADRS (2/19) (**Table 2**).

**Table 2:** MST treatment response rate and remission rate (N = 19).

Scale	Responders (n/N)	Response rate (%)	Remitters (n/N)	Remission rate (%)
HAMD	8/19	42.1	0/19	0
HAMA	8/19	42.1	1/19	5.3
MADRS	11/19	57.9	2/19	10.5

Response was defined as a ≥50% reduction from baseline; remission was defined as HAMD ≤ 7, HAMA ≤ 7, and MADRS ≤ 10.

### Behavioral data note

Although reaction times, accuracy, and Nogo commission errors were recorded during the Go/Nogo task, a technical storage error resulted in incomplete behavioral data for a subset of participants, precluding formal analysis of these measures. All subsequent EEG analyses (ERP, ERSP, microstates, and CFC) were based on the full, high-quality EEG dataset, which remained intact. The number of participants with usable behavioral data, the pre/post missing-data pattern, and the likely source of data loss are reported in the **Supplementary Material**. Consequently, we cannot determine whether theta changes were associated with improved inhibition, a speed–accuracy tradeoff, altered attention/engagement, or no behavioral change.

### ERP results

All EEG results are based on 18 participants. No significant pre- to post-treatment changes were observed in Go/Nogo-related ERP components, including N2, P3, N2d, and P3d amplitudes (all P > 0.05; |d| < 0.30). Grand-average ERP waveforms for each component are shown in **Supplementary Figure S1**-**S8**.

### ERSP results

At the uncorrected threshold, total Nogo-related theta-band activity at Fz (4–7 Hz, 0.16–0.40 s) was lower after MST, t(17) = −2.864, P = 0.011, d = −0.645; FCz theta (4–7 Hz, 0.10–0.42 s) was also lower, t(17) = −2.178, P = 0.044, d = −0.490. However, after Benjamini–Hochberg FDR correction within the prespecified ERSP family (all 10 predefined time-frequency windows), neither effect remained significant (Fz q = 0.108; FCz q = 0.219; **Table 3**). These ERSP findings are therefore reported as nominal, uncorrected, exploratory results rather than significant treatment-related effects. ERSP time-frequency maps and bar plots for the nominal Nogo-related theta effects are shown in **Figure 1**; results for the Go condition are presented in **Figure 2**.

**Table 3:** ERSP statistical results for all predefined time-frequency windows.

Name	tval	pval	FDR q	Cohend
Go-FZ-8~13Hz-0.16~0.70s	-0.419211626	0.680310845	0.895	-0.094373738
Go-FCZ-4~7Hz-0.12~0.25s	-1.67493093	0.112243168	0.374	-0.377063681
Go-FCZ-8~13Hz-0.16~0.70s	-0.096614018	0.924162805	0.924	-0.021749916
Go-PZ-8~13Hz-0.20~0.60s	0.290159792	0.775203586	0.895	0.065321343
Nogo-FZ-4~7Hz-0.16~0.40s	-2.863780499	**0.010756412**	0.108	-0.644699875
Nogo-FZ-8~13Hz-0.18~0.85s	-0.354535818	0.727297127	0.895	-0.079813789
Nogo-FCZ-4~7Hz-0.10~0.42s	-2.177659035	**0.043804985**	0.219	-0.490238843
Nogo-FCZ-8~13Hz-0.20~0.80s	-0.249975026	0.805602372	0.895	-0.056274874
Nogo-CZ-4~7Hz-0.16~0.40s	-0.857096612	0.403312743	0.895	-0.192951276
Nogo-CZ-8~13Hz-0.18~0.90s	-0.377792299	0.710260391	0.895	-0.085049341

p values are uncorrected two-sided paired-samples t-test p values. FDR q values were computed using the Benjamini–Hochberg procedure across all 10 predefined ERSP time-frequency windows. No ERSP window survived FDR correction (all q > 0.05). Bold values indicate statistically significant uncorrected p values (p < 0.05).

**Figure 1:**
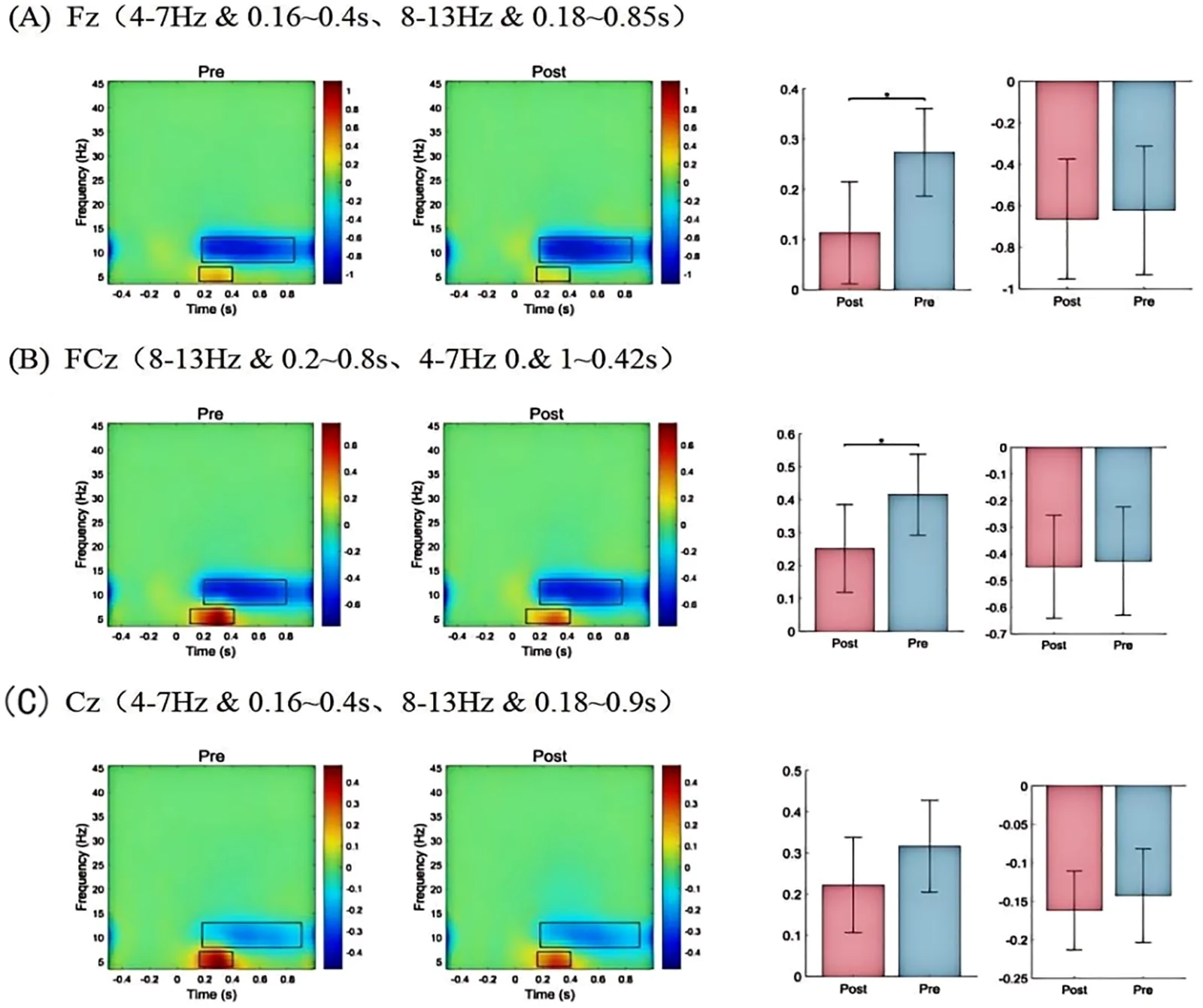
Microstate topography and GFP sequence for Go condition. Top: microstate maps (Map3, Map4, Map5) pre- and post-MST. Bottom: global field power (GFP) time course with color-coded microstate labeling. At the uncorrected threshold, Map4 AUC was lower and COG shifted later after MST; these effects did not survive FDR correction.

**Figure 2:**
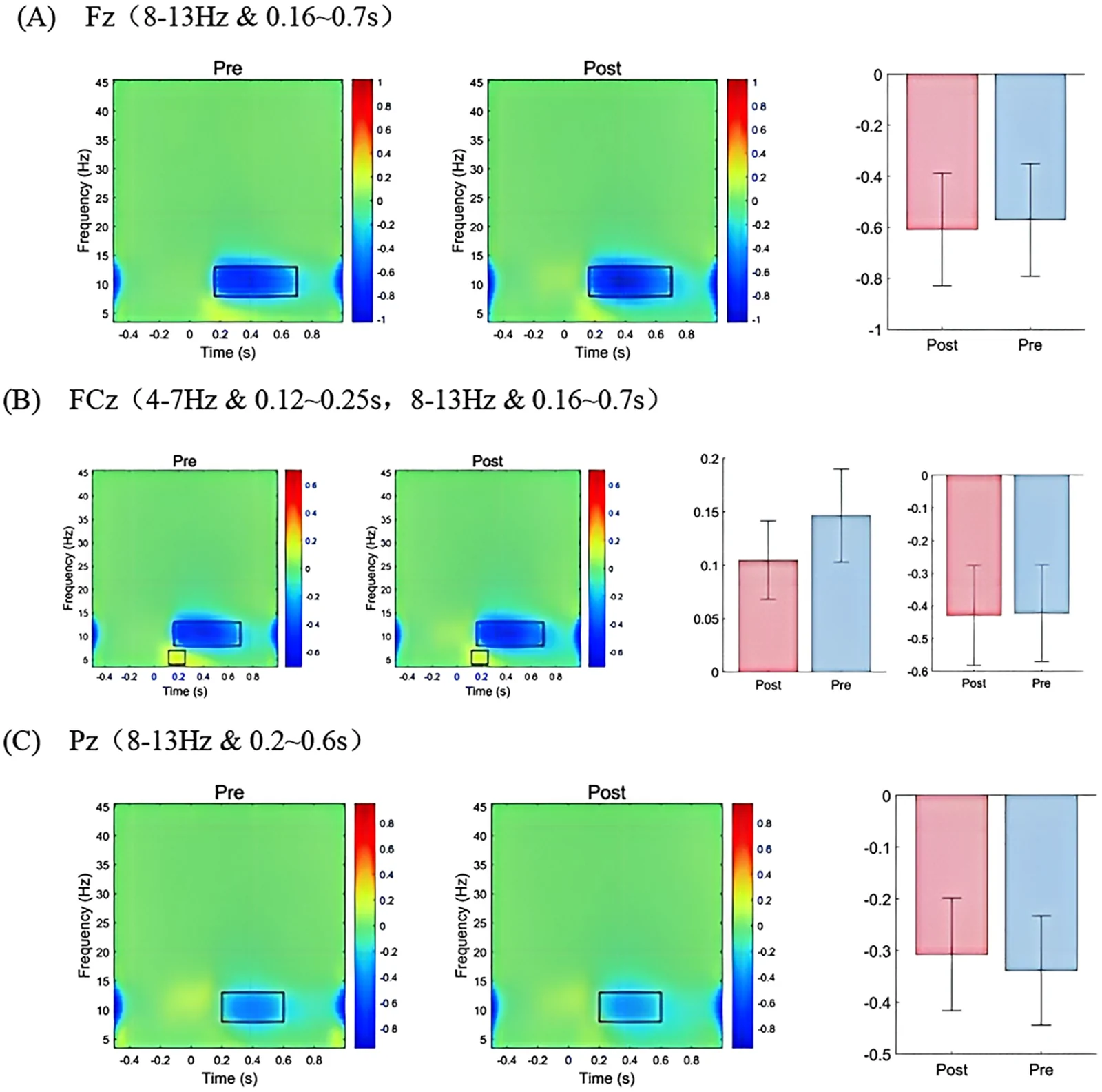
Microstate topography and GFP sequence for the Nogo condition.Top: microstate maps (Map2, Map3, Map5, Map6). Bottom: GFP time course. At the uncorrected threshold, Map2 duration was longer after MST; this effect did not survive FDR correction.

### Microstate results

In the Go condition, at the uncorrected threshold, Map4 AUC was lower after MST (pre: 33.1, post: 13.0 ms·μV, uncorrected P = 0.023) and Map4 COG shifted later (pre: 179.3, post: 198.4 ms, uncorrected P = 0.005; **Figure 3**; **Table 4**). In the Nogo condition, Map2 duration was longer after MST (pre: 46, post: 94 ms, uncorrected P = 0.025; **Figure 4**; **Table 5**). However, after Benjamini–Hochberg FDR correction within the microstate families, none remained significant (Go Map4 AUC q = 0.211; Go Map4 COG q = 0.095; Nogo Map2 duration q = 0.607). Therefore, all microstate findings are reported as nominal, uncorrected, exploratory results rather than significant treatment-related effects. No other Nogo-related microstate parameters changed significantly.

**Figure 3:**
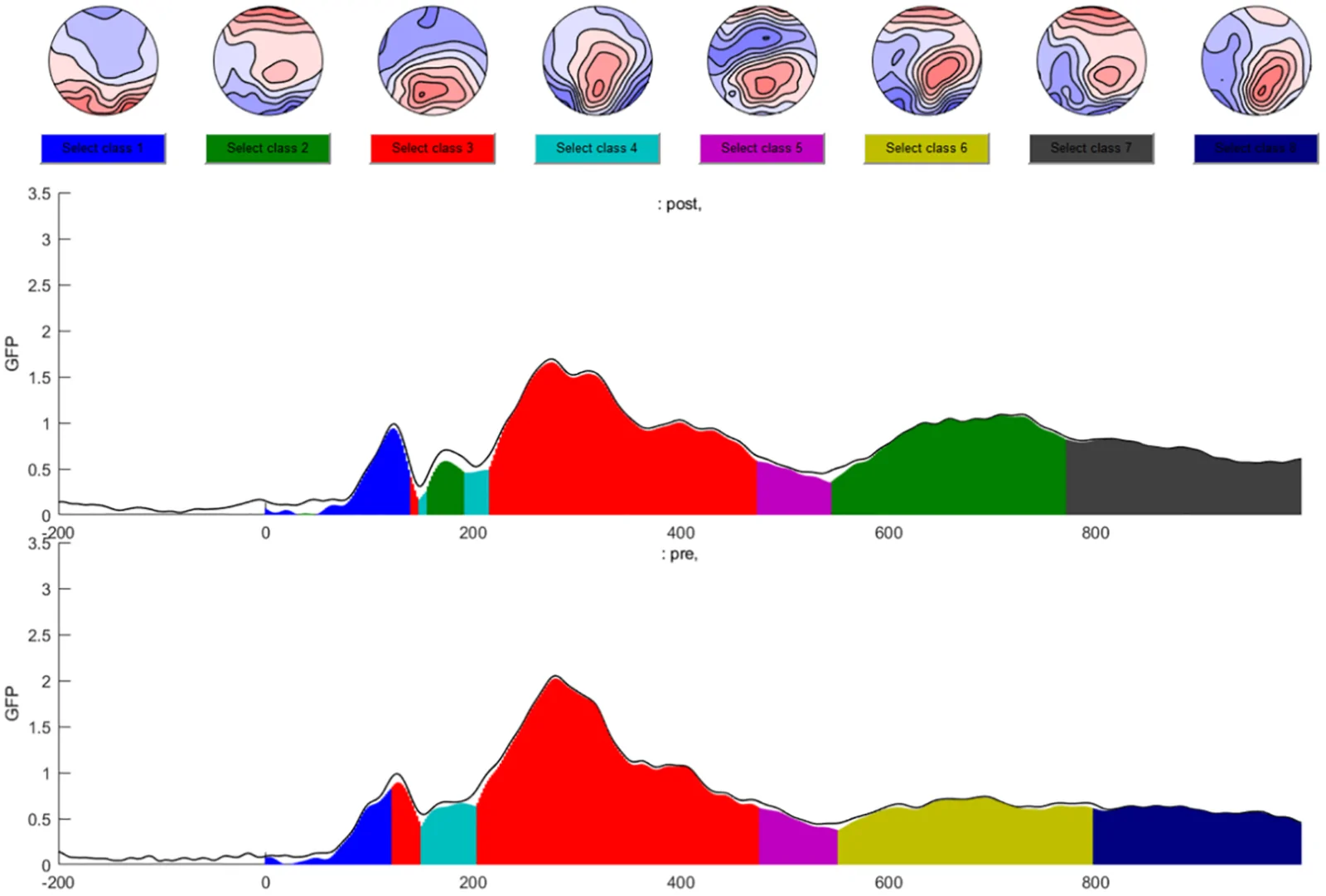
ERSP time-frequency plots and bar graphs for Nogo condition. **(A)** Fz (4–7 Hz, 0.16–0.40 s; 8–13 Hz, 0.18–0.85 s); **(B)** FCz (4–7 Hz, 0.10–0.42 s; 8–13 Hz, 0.20–0.80 s); **(C)** Cz (4–7 Hz, 0.16–0.40 s; 8–13 Hz, 0.18–0.90 s). Bar graphs show nominal, uncorrected lower total Nogo-Fz and Nogo-FCz theta-band activity (uncorrected P < 0.05; did not survive FDR correction).

**Figure 4:**
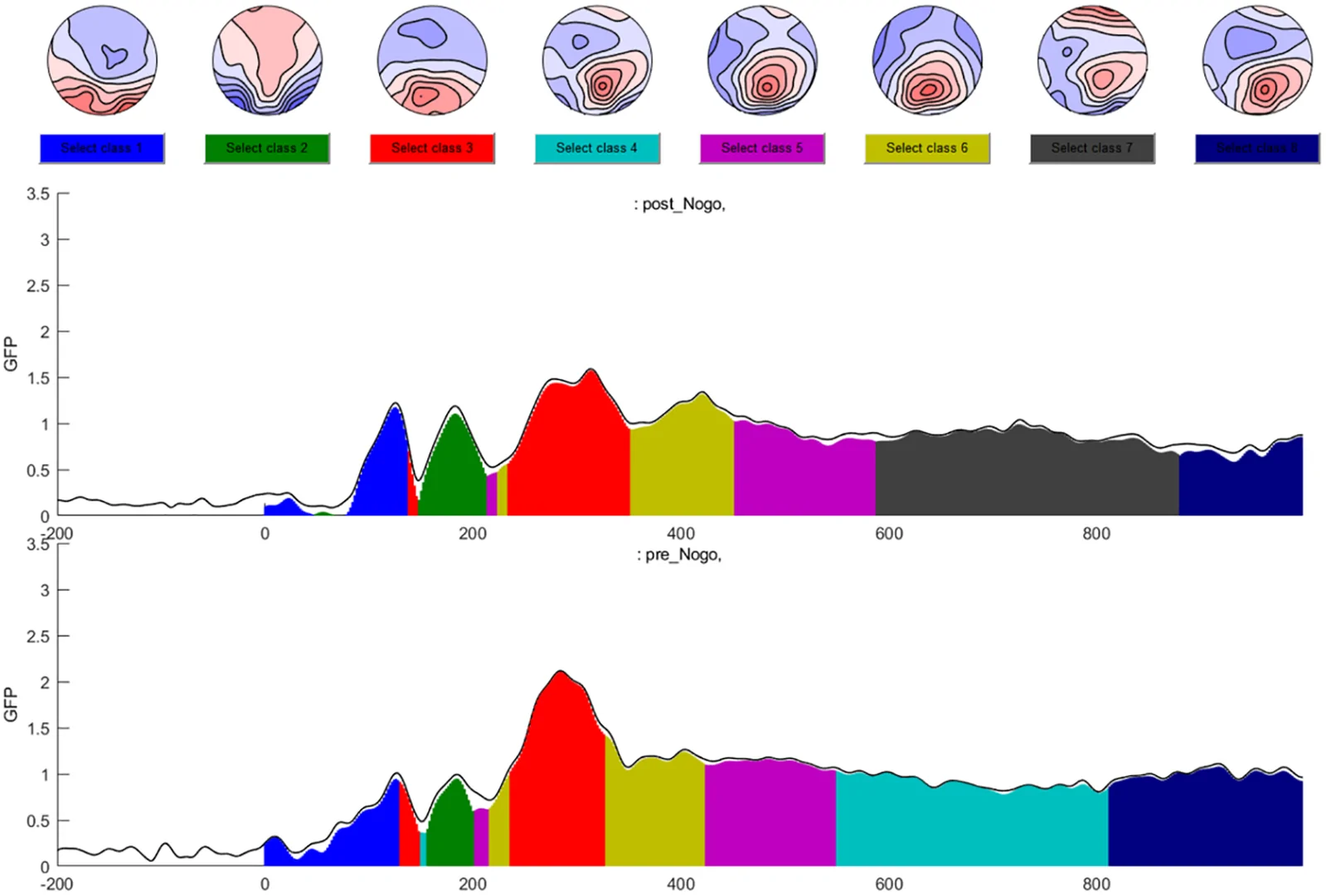
ERSP time-frequency plots and bar graphs for Go condition. **(A)** Time-frequency representation at Fz (8–13 Hz, 0.16–0.70 s); **(B)** at FCz (4–7 Hz, 0.12–0.25 s; 8–13 Hz, 0.16–0.70 s); **(C)** at Pz (8–13 Hz, 0.20–0.60 s). Right panels: bar graphs showing mean power. No significant changes.

**Table 4:** Microstate parameters in the Go condition before and after MST.

Microstate	Parameter	Post	Pre	p-value	FDR q
Map3	Onset(ms)	142	124	ns	ns
	Offset(ms)	474	476	ns	ns
	Duration(ms)	266	300	ns	ns
	Area under curve(AUC, ms ✖ μV)	292.2	357.9	ns	ns
	Center of gravity(COG, ms)	332.6	313.9	ns	ns
	Mean GFP(μV)	1.1289	1.2263	ns	ns
Map4	Onset(ms)	150	152	ns	ns
	Offset(ms)	216	204	ns	ns
	Duration(ms)	32	54	ns	ns
	Area under curve(AUC, ms ✖ μV)	13	33.1	0.0234	0.211
	Center of gravity(COG, ms)	198.4	179.3	0.0053	0.095
	Mean GFP(μV)	0.5093	0.665	ns	ns
Map5	Onset(ms)	476	478	ns	ns
	Offset(ms)	546	552	ns	ns
	Duration(ms)	72	76	ns	ns
	Area under curve(AUC, ms ✖ μV)	34.1	37.3	ns	ns
	Center of gravity(COG, ms)	508	511.5	ns	ns
	Mean GFP(μV)	0.5162	0.5267	ns	ns

Microstate parameters were compared between pre- and post-treatment using nonparametric randomization tests. Uncorrected p values are shown. FDR q values were computed using the Benjamini–Hochberg procedure within the Go microstate family (3 maps × 6 parameters = 18 tests). None of the Go microstate parameters survived FDR correction (all q > 0.05). “ns” indicates uncorrected p > 0.05.

**Table 5:** Microstate parameters in the Nogo condition before and after MST.

Microstate	Parameter	Post	Pre	p-value	FDR q
Map2	Onset(ms)	50	158	ns	ns
	Offset(ms)	214	202	ns	ns
	Duration(ms)	94	46	0.0253	0.607
	Area under curve(AUC, ms ✖ μV)	52	35.2	ns	ns
	Center of gravity(COG, ms)	182.7	181.2	ns	ns
	Mean GFP(μV)	0.6279	0.8507	ns	ns
Map3	Onset(ms)	140	132	ns	ns
	Offset(ms)	352	328	ns	ns
	Duration(ms)	128	112	ns	ns
	Area under curve(AUC, ms ✖ μV)	150.2	172.4	ns	ns
	Center of gravity(COG, ms)	292.8	273.7	ns	ns
	Mean GFP(μV)	1.2138	1.5698	ns	ns
Map5	Onset(ms)	216	204	ns	ns
	Offset(ms)	588	550	ns	ns
	Duration(ms)	146	140	ns	ns
	Area under curve(AUC, ms ✖ μV)	125.2	149.4	ns	ns
	Center of gravity(COG, ms)	506.8	471.2	ns	ns
	Mean GFP(μV)	0.9005	1.1022	ns	ns
Map6	Onset(ms)	226	218	ns	ns
	Offset(ms)	452	424	ns	ns
	Duration(ms)	110	116	ns	ns
	Area under curve(AUC, ms ✖ μV)	117.4	129.1	ns	ns
	Center of gravity(COG, ms)	397.2	358.3	ns	ns
	Mean GFP(μV)	1.1002	1.1434	ns	ns

Microstate parameters were compared between pre- and post-treatment using nonparametric randomization tests. Uncorrected p values are shown. FDR q values were computed using the Benjamini–Hochberg procedure within the Nogo microstate family (4 maps × 6 parameters = 24 tests). None of the Nogo microstate parameters survived FDR correction (all q > 0.05). “ns” indicates uncorrected p > 0.05.

### CFC results

No significant pre- to post-treatment change was found in theta-beta cross-frequency coupling (Fz theta–Cz beta) during the Nogo condition, *t*(17) = –0.557, *P* = 0.585, Cohen’s *d* = –0.125).

## Discussion

The present study examined clinical and task-evoked EEG changes following a course of MST in patients with TRD. The main findings were threefold. First, patients showed significant short-term reductions in depressive and anxiety symptoms. Second, at an uncorrected threshold, total Nogo-related theta-band activity at Fz and FCz was lower from pre- to post-treatment, whereas no such change was observed in the Go condition; however, these effects did not survive FDR correction, and because a Time × Condition interaction was not tested, condition specificity cannot be claimed. Third, exploratory microstate analysis showed nominal changes in Map4 AUC, Map4 COG, and Map2 duration, none of which survived FDR correction. In contrast, conventional Go/Nogo ERP amplitudes (N2, P3, N2d, and P3d) did not change significantly, and theta–beta cross-frequency coupling in the Nogo condition remained unchanged. The different baseline windows for ERP and ERSP may have contributed to the observed ERP–ERSP dissociation; sensitivity analyses using alternative baselines were not performed. Together, these results indicate that MST was associated with lower total Nogo-related theta-band activity at an uncorrected threshold, but this effect did not survive FDR correction. Because behavioral data were incomplete and no normative comparison was performed, the functional significance of this reduction remains uncertain, and the findings should be regarded as exploratory and hypothesis-generating.

MST produced substantial short-term clinical improvement. Scores on all three clinical scales decreased significantly after treatment, with large within-subject effect sizes. These findings are consistent with evidence that MST exerts clinically meaningful antidepressant effects in severe and treatment-resistant mood disorders (35). Although response rates were moderate to high, remission rates remained relatively low, suggesting robust acute symptom reduction without complete symptomatic recovery in most patients. Several factors may account for this. First, patients had severe baseline symptoms (mean HAMD-24 > 40), indicating a highly symptomatic TRD sample. Second, the average number of MST sessions was 8.2, which may be insufficient for full remission in some patients. Third, outcomes were assessed within 24–48 hours after the final treatment, capturing short-term rather than delayed or sustained recovery. Thus, the clinical findings should be interpreted as evidence of significant acute improvement rather than definitive remission.

The most prominent nominal EEG finding was lower total Nogo-related theta-band activity after MST at the uncorrected threshold; however, it did not survive FDR correction. Frontal midline theta is linked to conflict monitoring, response inhibition, and cognitive-control recruitment within fronto-cingulate circuits, with higher Nogo-related theta indicating greater ACC–dmPFC engagement (18, 19). In severe or treatment-resistant depression, exaggerated task-related theta may reflect inefficient or compensatory recruitment rather than optimal control (36). The observed reduction may reflect a change in task-related neural recruitment, but its functional significance remains uncertain because no normative comparison, behavioral data, Time × Condition interaction, or EEG-clinical association analysis was available. Whether baseline theta was abnormal or post-treatment theta approached normative levels cannot be determined. Frontal midline theta can index conflict monitoring, control recruitment, task engagement, effort, arousal, or performance differences, and a reduction is not inherently beneficial (18–20). Therefore, the reduction should not be equated with improved neural efficiency or improved response inhibition.

Although theta was lower in the Nogo but not the Go condition at the uncorrected threshold, a formal Time × Condition interaction was not tested; therefore, condition specificity cannot be claimed. This pattern is consistent with the possibility that MST-related modulation was most evident under high inhibitory-control demands, but this remains a hypothesis for future studies with direct interaction tests. Similarly, frontal-midline specificity is limited to the *a priori* electrodes Fz and FCz.

One possible mechanism is that MST alters the excitability and network dynamics of corticothalamic and fronto-limbic circuits through repeated seizure induction. Within this framework, lower total theta-band activity may reflect a change in task-related neural activity, but this remains speculative and cannot be interpreted as improved neural efficiency. The absence of significant N2/P3 changes suggests that any pre–post change was not evident in conventional phase-locked ERP amplitudes. However, because the theta measure reflects total theta-band activity and did not survive FDR correction, this dissociation should be interpreted cautiously and cannot be taken as evidence that MST preferentially modulates oscillatory network coordination.

The microstate findings were exploratory and nominal; none survived FDR correction. They may suggest changes in the temporal organization of task-evoked brain states, but cannot be considered convergent evidence for a neural-efficiency account. In the Go condition, Map4 (associated with early attentional orienting and motor preparation) showed a nominal reduction in AUC and a later center of gravity after MST, suggesting modified expression and temporal distribution of this task-related brain state. In the Nogo condition, Map2 (possibly reflecting preparatory inhibitory state) showed a nominal prolonged duration, indicating a more sustained brain state during response inhibition. Together, these findings suggest that MST may influence the millisecond-level temporal organization of neural processing during response execution and inhibition. However, microstate maps are data-driven and task-dependent, and microstate numbering is dataset-dependent; functional assignments should be considered tentative (26). We did not directly test whether microstate changes were related to theta-band changes, so these findings cannot be considered convergent evidence for a neural-efficiency account. They are reported as complementary, hypothesis-generating observations requiring replication and direct testing.

Nogo-related frontal theta-band activity may warrant investigation as a candidate exploratory correlate in future studies, but the present design cannot support biomarker utility, monitoring, dose adjustment, or closed-loop claims. Because EEG was assessed only before and after treatment and we did not test whether theta changes covaried with symptom changes, the two-time-point design cannot support real-time monitoring, session-by-session dose adjustment, or closed-loop MST. These remain future directions requiring adequately powered longitudinal studies with behavioral and clinical outcome measures.

Several limitations should be acknowledged. Because this was an open-label, single-arm, pre–post study, observed changes cannot be attributed specifically to MST. Practice effects, regression to the mean, symptom fluctuation, expectancy, concomitant care, anesthesia, seizure-related nonspecific effects, and medication effects cannot be excluded.

Behavioral data were incomplete due to a technical storage error; therefore, reaction times, accuracy, and Nogo commission errors could not be formally analyzed, and we cannot determine whether theta changes were associated with improved inhibition, a speed–accuracy tradeoff, altered attention/engagement, or no behavioral change. FDR correction was applied within prespecified ERSP and microstate families; however, no EEG finding survived correction. The nominal theta effects were limited to the *a priori* electrodes Fz and FCz. For microstate analysis, the group-level template may obscure individual topographic differences, and its application to task-evoked data remains debated (26); therefore, these microstate findings should be considered preliminary.

The asymmetric assessment timing (baseline ≤24 h pre-MST; post-treatment 24–48 h post-MST) was chosen to avoid acute postictal effects, but residual postictal EEG slowing may still have been present at the post-treatment recording and could have influenced spectral power, including theta-band activity, as well as microstate dynamics. Regarding anesthesia, propofol was used for induction in all MST sessions and may influence seizure duration and ictal EEG expression. Although propofol and succinylcholine have short half-lives, cumulative anesthesia exposure cannot be fully ruled out (37). Because propofol was administered according to a standard protocol, it is unlikely to explain the within-subject pre–post changes, but it may still contribute to inter-individual variability in seizure duration and task-evoked EEG measures.

In addition, benzodiazepines, lithium, and anticonvulsants can affect seizure threshold, seizure duration, and EEG spectra. In this sample, several patients received lorazepam, clonazepam, oxazepam, eszopiclone, lithium carbonate, oxcarbazepine, lamotrigine, or sodium valproate (**Supplementary Table S1**). These medications, together with the standardized anesthetic protocol, may have influenced seizure duration, ictal expression, and post-treatment EEG measures. Importantly, the effects of anticonvulsants and anesthetic agents such as propofol on ERSP, including theta-band activity, remain poorly understood and warrant further investigation.

Although bipolar disorder was an exclusion criterion, mood stabilizers were widely used in this cohort. In patients with TRD, mood stabilizers such as lithium, lamotrigine, valproate, and oxcarbazepine are commonly prescribed as adjunctive treatments to augment antidepressant efficacy and control agitation, irritability, or anxious arousal associated with severe depression, rather than to treat bipolar disorder itself. Their use therefore does not necessarily indicate undiagnosed bipolar disorder. Medication regimens were kept stable for at least 2 weeks before baseline and throughout MST; thus, acute medication changes are unlikely to explain the observed pre–post changes. However, stable effects of concomitant medications on seizure threshold and EEG spectra cannot be excluded. No patient experienced intercurrent illness or medication changes during the study period.

Finally, the different baseline windows for ERP (−200 to 0 ms) and ERSP (−350 to −150 ms) may have contributed to the divergent results observed in this study; future studies should adopt a unified baseline window for consistent comparisons.

Despite these limitations, this exploratory study may still offer some preliminary observations. First, it moves beyond whether MST improves depressive symptoms to ask whether MST may be associated with changes in task-evoked cognitive-control circuits. By focusing on theta-band activity, it tentatively links a well-established marker of effortful control to clinical neuromodulation in TRD. Second, the pattern of unchanged ERP components alongside nominally lower total theta-band activity may be consistent with the possibility that MST-related changes are more readily detectable in time-frequency measures than in conventional time-domain ERP amplitudes, although this interpretation remains speculative because the theta effect did not survive FDR correction. Third, the combined ERSP and microstate findings are exploratory and nominal; they may help generate hypotheses about MST-related changes infronto-cingulate dynamics, but they cannot be considered convergent evidence. If replicated in larger controlled studies, Nogo-related theta activity might be examined as a candidate exploratory correlate. Biomarker utility and individualized neuromodulation strategies remain unproven. Future large-scale, multisite controlled trials incorporating source localization, longitudinal follow-up, and prospective suicide risk assessment are needed to test these preliminary observations and determine whether Nogo-related theta dynamics can predict clinical response, sustained remission, or anti-suicidal effects.

## Conclusions

In this exploratory, single-arm, pre–post study, a course of MST was associated with short-term reductions in symptom scores. At an uncorrected threshold, total Nogo-related frontal theta-band activity at Fz and FCz was lower after treatment; however, these effects did not survive FDR correction, and no Time × Condition interaction was tested. Therefore, condition specificity, causal mechanisms, improved neural efficiency, and biomarker utility cannot be claimed. These preliminary findings require replication in controlled studies with complete behavioral data, normative comparisons, and longitudinal clinical–EEG association analyses. Future large-scale, multisite trials incorporating source localization, longitudinal follow-up, and prospective suicide risk assessment are needed to determine whether Nogo-related theta dynamics can predict clinical response, sustained remission, or anti-suicidal effects.

## Data Availability

The original contributions presented in the study are included in the article/**Supplementary Material**. Further inquiries can be directed to the corresponding authors.

## Supplementary material

Supplementary Table 1 Participant-level demographic, clinical, MST treatment, anesthesia, and concomitant medication information.Mean age was 20.9 ± 8.2 years (range 13–40); 10/19 participants were minors.

Supplementary Table 2 Normality test results for seizure duration variables (Shapiro–Wilk test, N = 19).

Supplementary Figure 1 Go-N2 waveform at electrode Fz. Grand-averaged ERP waveforms pre- (blue) and post-MST (red). No significant difference.

Supplementary Figure 2 Go-N2 waveform at electrode FCz. No significant difference.

Supplementary Figure 3 Go-P3 waveform at electrode Pz. No significant difference.

Supplementary Figure 4 Nogo-N2 waveform at electrode Fz. No significant difference.

Supplementary Figure 5 Nogo-N2 waveform at electrode FCz. No significant difference.

Supplementary Figure 6 Nogo-P3 waveform at electrode Cz.No significant difference.

Supplementary Figure 7 N2d difference wave (Nogo minus Go) at electrode FCz. No significant difference.

Supplementary Figure 8 P3d difference wave (Nogo minus Go) at electrode Cz. No significant difference.
