# Supplementary material for: Nogo-related frontal theta-band activity after magnetic seizure therapy in treatment-resistant depression: an exploratory task-based EEG study
Source: Front Psychiatry. 2026 Oct 28;17:1950544. doi: 10.3389/fpsyt.2026.1950544 (PMC13752014; doi:10.3389/fpsyt.2026.1950544)
Supplement: Supplementary file 1 [file Supplementaryfile1.docx]

**Supplementary**

Table S1. Participant-level demographic, clinical, MST treatment, anesthesia, and concomitant medication information. Mean age was 20.9 ± 8.2 years (range 13–40); 10/19 participants were minors.

| ID | Sex | Age | Session | Weight (kg) | Propofol  (mg) | Succinylcholine  (mg) | Medication |
| --- | --- | --- | --- | --- | --- | --- | --- |
| 1 | F | 25 | 12 | 48 | 60 | 40 | Lamotrigine 125 mg qd;  lorazepam 0.5 mg qn;  trazodone 50 mg qn;  aripiprazole 5 mg qd;  bupropion 150 mg qd; tandospirone 10 mg tid;  duloxetine 60 mg qd |
| 2 | F | 15 | 6 | 40 | 55 | 35 | Fluoxetine 30 mg qd;  alprazolam 0.4 mg qn |
| 3 | M | 40 | 6 | 80 | 70 | 50 | Quetiapine 150 mg qn; sertraline 50 mg bid; lorazepam 0.25 mg bid; buspirone 15 mg tid; eszopiclone 2 mg qn; trazodone 12.5 mg qn |
| 4 | F | 15 | 8 | 50 | 50 | 40 | Magnesium valproate SR 250 mg bid; aripiprazole 5 mg qn; escitalopram 15 mg qd; lorazepam 0.75 mg qn |
| 5 | F | 19 | 8 | 42 | 45 | 30 | Tandospirone 10 mg tid; aripiprazole 5 mg qd; sertraline 100 mg qd; clonazepam 1 mg qn |
| 6 | F | 14 | 10 | 76 | 120 | 50 | Aripiprazole 5 mg qn; escitalopram 15 mg qd; lamotrigine 25 mg bid; lithium carbonate 500 mg qn |
| 7 | F | 15 | 8 | 65 | 55 | 40 | Oxcarbazepine 300 mg bid;  clonazepam 2 mg qn; trazodone 25 mg qn; aripiprazole 5 mg qd |
| 8 | F | 16 | 8 | 40 | 40 | 30 | Olanzapine 5 mg qn; escitalopram 20 mg qd |
| 9 | F | 22 | 8 | 62 | 60 | 50 | Aripiprazole 2.5 mg qn; alprazolam 0.6 mg qn; escitalopram 20 mg qd |
| 10 | M | 15 | 6 | 85 | 90 | 60 | Fluoxetine 20 mg qd; lithium carbonate 300 mg bid; alprazolam 0.8 mg qn; aripiprazole 5 mg qd |
| 11 | F | 25 | 12 | 50 | 50 | 40 | Lithium carbonate 300 mg bid; quetiapine 300 mg qn; zolpidem 10 mg qn; fluoxetine 20 mg qd |
| 12 | F | 19 | 8 | 65 | 50 | 40 | Lithium carbonate 300 mg bid; quetiapine 400 mg qn; lorazepam 0.75 mg qn; eszopiclone 3 mg qn |
| 13 | F | 36 | 7 | 55 | 50 | 40 | Aripiprazole 5 mg bid; sertraline 150 mg qd; lorazepam 0.5 mg qn |
| 14 | F | 16 | 8 | 60 | 60 | 45 | Duloxetine 20 mg qn; quetiapine 200 mg qn; aripiprazole 5 mg bid; lorazepam 0.5 mg qn; oxcarbazepine 300 mg qam and 600 mg qpm |
| 15 | F | 17 | 8 | 67 | 70 | 50 | Aripiprazole 5 mg qn; lithium carbonate 300 mg qn; oxazepam 7.5 mg qn |
| 16 | M | 35 | 7 | 52 | 65 | 45 | Lamotrigine 12.5 mg qd; quetiapine 100 mg qd and 200 mg qn |
| 17 | F | 13 | 8 | 63 | 60 | 45 | Eszopiclone 3 mg qn; vortioxetine 15 mg qd; aripiprazole 7.5 mg qd |
| 18 | F | 25 | 8 | 90 | 80 | 50 | Bupropion 150 mg qd; sodium valproate 500 mg bid; clonazepam 1 mg qn; quetiapine 50 mg qn; aripiprazole 5 mg qd |
| 19 | F | 15 | 8 | 70 | 60 | 35 | Quetiapine 150 mg qn; escitalopram 15 mg qd; lorazepam 0.5 mg qn |

**Note:**MST, magnetic seizure therapy; qd, once daily; bid, twice daily; tid, three times daily; qn, at bedtime; qam, every morning; qpm, every evening. Propofol and succinylcholine doses are per MST session. Participant IDs were recoded to reduce re-identification risk; original IDs are not provided.

Table S2. Normality test results for seizure duration variables (Shapiro–Wilk test, N = 19)

| Variable name | **n** | Mean ± SD | Skewness | **W** | p | Normal distribution |
| --- | --- | --- | --- | --- | --- | --- |
| Mean seizure duration | 19 | 13.73 ± 2.12 | 0.680 | 0.9497 | 0.3901 | Yes |
| Median seizure duration | 19 | 13.13 ± 1.68 | 0.597 | 0.9298 | 0.1715 | Yes |
| SD of seizure duration | 19 | 2.94 ± 1.83 | 1.018 | 0.9031 | 0.0555 | Yes |
| First-session seizure duration | 19 | 13.26 ± 3.23 | 0.986 | 0.8959 | 0.0410 | No |
| Last-session seizure duration | 19 | 14.63 ± 4.71 | 1.645 | 0.7656 | 0.0004 | No |
| Total seizure duration | 19 | 111.21 ± 28.26 | 0.857 | 0.8761 | 0.0183 | No |
| Slope of seizure duration | 19 | 0.11 ± 0.60 | -0.470 | 0.9705 | 0.7871 | Yes |
| Last minus first session difference | 19 | 1.37 ± 5.60 | 0.694 | 0.8831 | 0.0243 | No |

**Note:** Seizure duration is expressed in seconds (s). Normality was assessed using the Shapiro–Wilk test; *p* > 0.05 indicates a normal distribution. SD, standard deviation; W, Shapiro–Wilk statistic. For sz_sd, *p* = 0.056, which is borderline but above the 0.05 threshold.

Figure S1. Go-N2 waveform at electrode Fz. Grand-averaged ERP waveforms pre- (blue) and post-MST (red). No significant difference.

Figure S2. Go-N2 waveform at electrode FCz. No significant difference.

Figure S3. Go-P3 waveform at electrode Pz. No significant difference.

Figure S4. Nogo-N2 waveform at electrode Fz. No significant difference.

Figure S5. Nogo-N2 waveform at electrode FCz. No significant difference.

Figure S6. Nogo-P3 waveform at electrode Cz.No significant difference.

Figure S7. N2d difference wave (Nogo minus Go) at electrode FCz. No significant difference.

Figure S8. P3d difference wave (Nogo minus Go) at electrode Cz. No significant difference.

Supplementary Behavioral Data Note

Although reaction times, accuracy, and Nogo commission errors were recorded during the Go/Nogo task, a technical storage error during data export resulted in incomplete behavioral data. Usable pre-treatment behavioral data were available for 10/19 participants, usable post-treatment behavioral data for 16/19, and paired pre-post behavioral data for 10/19 participants. Missingness occurred at the file-export stage and was not related to clinical or EEG variables. Because paired behavioral data were incomplete, these measures were not formally analyzed. All subsequent EEG analyses (ERP, ERSP, microstates, and CFC) were based on the full, high-quality EEG dataset, which remained complete for 18 participants. Consequently, we cannot determine whether theta changes were associated with improved inhibition, a speed-accuracy tradeoff, altered attention/engagement, or no behavioral change.
